# Clothianidin-resistant *Anopheles gambiae* adult mosquitoes from Yaoundé, Cameroon, display reduced susceptibility to SumiShield® 50WG, a neonicotinoid formulation for indoor residual spraying

**DOI:** 10.1186/s12879-024-09030-8

**Published:** 2024-01-25

**Authors:** Caroline Fouet, Fred A. Ashu, Marilene M. Ambadiang, Williams Tchapga, Charles S. Wondji, Colince Kamdem

**Affiliations:** 1 Department of Biological Sciences, The University of Texas at El Paso, 500 W. University Ave, El Paso, TX 79968 USA; 2 Centre for Research in Infectious Diseases (CRID), P.O. Box 13591, Yaoundé, Cameroon; 3 Department of Biochemistry, Faculty of Science, University of Yaoundé 1, P.O. Box 812, Yaoundé, Cameroon; 4 Vector Biology Department, Liverpool School of Tropical Medicine (LSTM), Pembroke Place, Liverpool, L3 5QA UK

**Keywords:** *Anopheles*, Clothianidin, Resistance, SumiShield® 50WG, Malaria, Neonicotinoids

## Abstract

**Background:**

Chronic exposure of mosquito larvae to pesticide residues and cross-resistance mechanisms are major drivers of tolerance to insecticides used for vector control. This presents a concern for the efficacy of clothianidin, an agricultural neonicotinoid prequalified for Indoor Residual Spraying (IRS).

**Methods:**

Using standard bioassays, we tested if reduced susceptibility to clothianidin can affect the efficacy of SumiShield® 50WG, one of four new IRS formulations containing clothianidin. We simultaneously monitored susceptibility to clothianidin and to SumiShield 50WG, testing adults of *Anopheles gambiae*, *An. coluzzii* and *Culex* sp sampled from urban, suburban and agricultural areas of Yaoundé, Cameroon.

**Results:**

We found that in this geographic area, the level of susceptibility to the active ingredient predicted the efficacy of SumiShield 50WG. This formulation was very potent against populations that reached 100% mortality within 72 h of exposure to a discriminating concentration of clothianidin. By contrast, mortality leveled off at 75.4 ± 3.5% within 7 days of exposure to SumiShield 50WG in *An. gambiae* adults collected from a farm where the spraying of the two neonicotinoids acetamiprid and imidacloprid for crop protection is likely driving resistance to clothianidin.

**Conclusions:**

Despite the relatively small geographic extend of the study, the findings suggest that cross-resistance may impact the efficacy of some new IRS formulations and that alternative compounds could be prioritized in areas where neonicotinoid resistance is emerging.

## Background

Over the past two decades, malaria prevention in sub-Saharan Africa has relied on two core interventions which used chemical insecticides [1]. Indoor residual spraying (IRS) and long-lasting insecticidal nets (LLINs) have contributed to a substantial reduction in the disease burden, but the Achilles’ heel of both intervention measures is the development of insecticide resistance among vector populations [2, 3]. A limited number of active ingredients satisfy the criteria to be safely and effectively deployed on a large scale in endemic areas [4]. The spread of insecticide resistance poses a major challenge not only to the efficacy of IRS and LLINs, but also to their cost-effectiveness [5]. Control programs have often attempted to mitigate the negative impacts of insecticide resistance by switching between active ingredients [6]. For example, some endemic countries have progressively adopted more expensive alternatives such as carbamates and organophosphates for IRS to mitigate mosquito resistance to pyrethroids. This shift has been responsible for a decline in IRS coverage from 5% in 2010 to 2% in 2018 [7]. Sequential deployment of insecticides is also associated with the development of multiple forms of phenotypic, genetic and behavioral resistance that quickly become established in wild mosquito populations [1]. In order to reduce the likelihood of resistance development, the WHO’s global plan for insecticide resistance management in malaria vectors has recommended using active ingredients in rotations, combinations, mosaics and mixtures [1, 8]. However, for this management strategy to be successful, it is imperative to reinforce rotation programs with new chemicals which remain effective against mosquito populations that are tolerant to existing insecticides [4]. To identify these alternatives, a number of agrochemicals have been tested and a few candidates have been selected for use in malaria vector control [9–13].

Clothianidin is used in four new IRS formulations that have been prequalified by the World Health Organization (WHO) [4]. Two formulations contain clothianidin alone: SumiShield 50WG developed by Sumitomo Chemical and Klypson 500 WG (Tagros Chemicals India Pvt. Ltd). Two other products (Fludora Fusion (Bayer Environmental Science) and 2GARD (Tagros Chemicals India Pvt. Ltd)) contain a mixture of clothianidin and deltamethrin [14, 15]. Clothianidin belongs to a class of 8 registered chemicals known as neonicotinoids [16, 17]. Neonicotinoids which have become the most widely used agricultural pesticides worldwide target the nicotinic acetylcholine receptor (nAChR) in the insect central nervous system and cause over-stimulation, which may result in paralysis and death [17]. Although neonicotinoids are neurotoxic insecticides, their mode of action is sufficiently distinct, thus limiting the risk of cross-resistance with other neurotoxic chemicals such as pyrethroids widely used in malaria prevention.

Field trials of IRS formulations containing clothianidin have revealed long-lasting insecticidal activity against different vector species on diverse surfaces [18–22]. Thus, clothianidin is considered a promising alternative to control malaria vectors in areas with high pre-existing resistance to multiple insecticide classes. In preparation for rollout of clothianidin formulations as part of national IRS rotation strategies, the U.S. President’s Malaria Initiative has conducted susceptibility testing in anopheline populations from 16 African countries [23]. This investigation suggested that most populations of the major vectors of *Plasmodium* parasites across the continent were susceptible to filter papers impregnated with SumiShield 50WG at a diagnostic concentration of 2% (w/v) clothianidin. While the findings of this study are encouraging, targeted testing of populations from agricultural regions where *Anopheles* mosquitoes are more likely to develop resistance to neonicotinoids should be conducted alongside continental-scale surveys.

The role of agricultural pesticides spraying in the development of resistance to insecticides used in malaria mosquito control has been widely documented [24–31]. It has recently been observed that some larval populations of *An. gambiae* can grow and emerge in water containing doses of neonicotinoids that are lethal to susceptible strains [32]. This finding suggested that exposure to pesticide residues and/or other cross-resistance mechanisms could be selecting for neonicotinoid tolerance in *An. gambiae*. Indeed, susceptibility tests have revealed resistance to thiamethoxam, imidacloprid and acetamiprid in some adult populations of *An. gambiae* and *An. coluzzii* [33–35]. Neonicotinoids are highly soluble in water and can persist for months in aerobic soils, therefore making contamination of standing waters, which serve as mosquito breeding sites in farming areas very likely [36–38]. Published information on the use of neonicotinoids across Sub-Saharan Africa is lacking, but preliminary reports from Cameroon, Tanzania and Ivory Coast suggested that hundreds of commercial formulations of neonicotinoids have been registered for use in crop protection [39–42].

In addition to residual pesticide exposure, other cross-resistance mechanisms could also contribute to increase the tolerance of anopheline populations to neonicotinoids. Notably, some detoxification enzymes could enhance metabolic resistance to neonicotinoids and reduced the baseline susceptibility in some species or populations. For instance, some populations of *An. funestus* whose larval habitats are less prone to residual pesticide contamination have been shown to resist a discrimination concentration of 150 µg/ml of clothianidin in laboratory bioassays [43]. Such findings highlight the importance of addressing cross-resistance and particularly the role of detoxification enzymes in the development of neonicotinoid tolerance in *Anopheles* mosquitoes. Here we conducted intensive testing of *Anopheles* and *Culex* adult mosquito populations from six locations including one of the largest urban farms in Cameroon, using standard bioassays. Our aim was to test if neonicotinoid tolerance observed in *An. gambiae* larvae and adults could affect the efficacy of SumiShield 50WG. We found that as previously demonstrated by continent-wide surveys [23], SumiShield 50WG is very effective against clothianidin-susceptible populations of *An. coluzzii* and *An. gambiae*. However, its efficacy is significantly reduced in *An. gambiae* adults displaying low mortality to clothianidin (150 µg/ml). These findings suggest that the efficacy of clothianidin and its formulations may be affected by susceptibility variations between species and populations. Populations from agricultural settings have a higher risk of developing resistance, and alternative chemicals could be prioritized in areas where tolerance to the active ingredient is observed.

## Methods

### Study sites

**Fig. 1:**
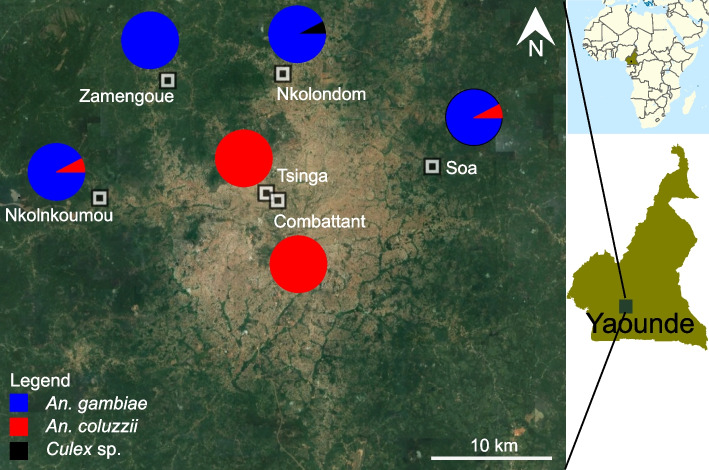
Map of the sampling locations where *Anopheles* and *Culex* mosquitoes were collected to evaluate their susceptibility to clothianidin and to SumiShield 50WG. The city of Yaoundé (brown areas) is surrounded by degraded forests

**Fig. 2:**
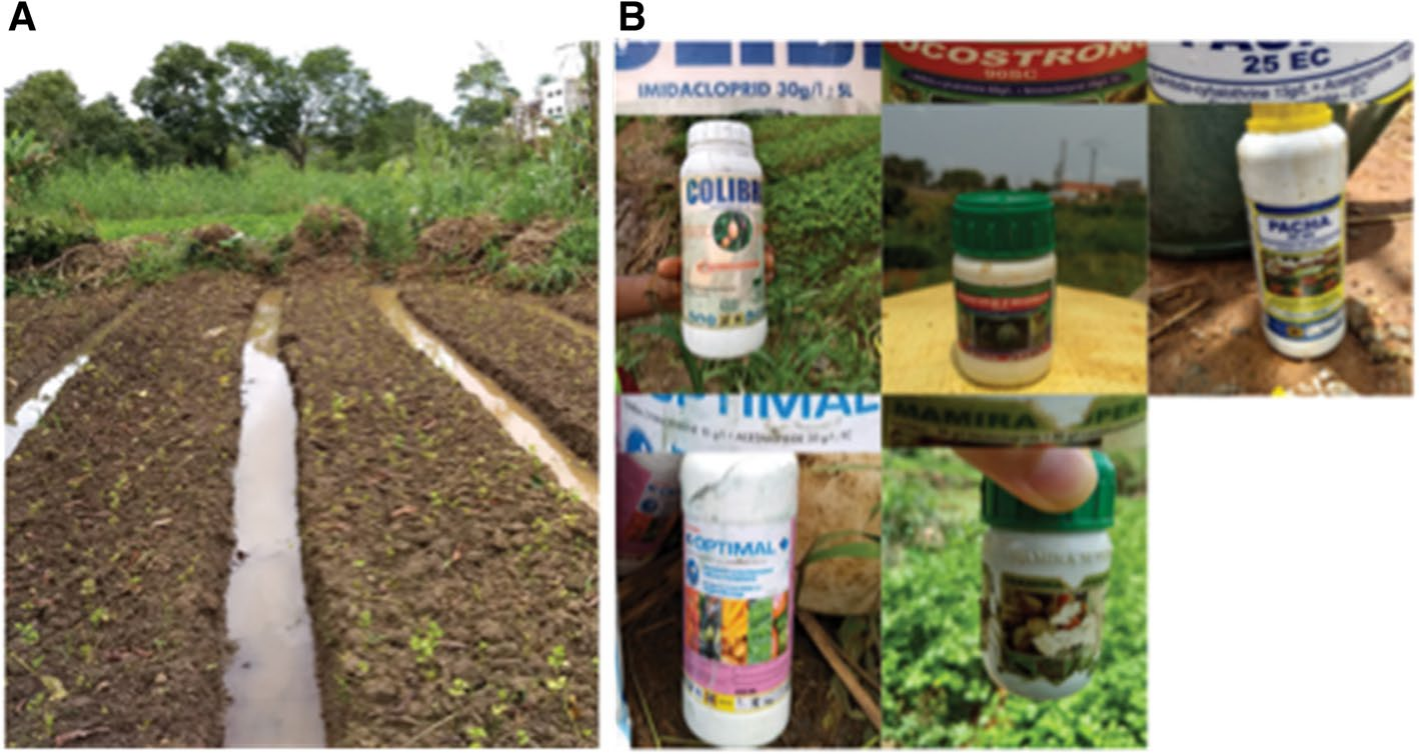
Picture of a larval breeding site in Nkolondom (**A**) and samples of empty containers found in the farm indicating the use of formulations of imidacloprid and acetamiprid (**B**)

We collected mosquitoes from four suburban sites in the outskirts of Yaoundé, the capital of Cameroon (Fig. 1). We also tested samples from two densely urbanized areas within the city. The suburban sites included Nkolondom (3°56′43" N, 11°31′01" E), situated approximately 7 km west of Yaoundé. Since 1985, a swampy area in Nkolondom is exploited for intensive crop cultivation [44]. In 2020, the farm which attracted at least 100 workers was subdivided into 100–200 mosaics of ~ 20m^2^ adjacent plots dedicated to the cultivation of aromatic herbs, amaranth and lettuce. Standing water between ridges and furrows provide mosquito breeding sites, which maintain large larval populations of *An. gambiae* and *Culex* sp throughout the year (Fig. 2A) [32–34].

### Mosquito populations

We sampled and tested mosquitoes from the six above-mentioned sites between 2019 and 2020. We collected larvae that were reared to adults and tested with standard bioassays. Larval breeding sites such as ephemeral or semi-permanent standing water created by the rain and human activities were identified and immature stages were sampled using dippers and transported in plastic containers to the insectary. In urban and suburban areas of Yaoundé, such aquatic larval habitats typically harbored immature stages of *An. gambiae *sensu lato ( *s.l.*) (i.e., the *An. gambiae* complex) and *Culex* sp. *An. gambiae s.l*. and *Culex* sp larvae were identified using morphological identification keys [45–48]. Larvae were reared to adults under standard laboratory conditions of 25–27°C, 70–90% relative humidity and a 12:12 h light/dark photoperiod [49]. The two sibling species of the *An. gambiae* complex: *An. gambiae *sensu stricto (hereafter referred to as *An. gambiae*) and *An. coluzzii* are the dominant malaria vectors in Yaoundé and in surrounding rural areas [50, 51]. *An. coluzzii* is found exclusively in the most urbanized neighborhoods while *An. gambiae* is the only species present in neighboring rural settings. To identify which species between *An. gambiae* and *An. coluzzii* was collected from each site, we genotyped 50 mosquito samples using a diagnostic PCR [52]. This method allowed us to identify species of the *An. gambiae* complex based on point mutations on the ribosomal DNA using PCR amplification and restriction digestion of amplicons. *Culex* sp larvae that occur in the same breeding sites as *An. gambiae* in the Nkolondom farm were also sampled, reared to adults and tested.

### Clothianidin susceptibility testing

The susceptibility of adult mosquitoes was tested against clothianidin using CDC bottle assays [53]. The bioassay procedure followed a modified version of the WHO standard operating procedure for testing the susceptibility of adult mosquitoes to clothianidin [54, 55]. Precisely, we did not used the vegetable oil ester, Mero®, as a surfactant. Recent studies showed that some vegetable oil-based surfactants such as Mero can enhance the toxicity of neonicotinoids leading to an overestimation of the insecticidal activity of the active ingredient [34, 43, 56]. Instead, mortality was evaluated against clothianidin alone dissolved in ethanol using a discriminating dose (i.e., the lowest dose at which 100% of adults from a susceptible population die) of 150 µg/ml as determined by a previous study [57]. We prepared stock solutions using a technical-grade formulation of clothianidin (PESTANAL®, analytical standard, Sigma-Aldrich, Dorset, United Kingdom) and absolute ethanol as solvent. The solutions were stored at 4°C for at least 24 h before use to maximize the solubility of clothianidin.

To perform bottle bioassays, each Wheaton 250-ml bottle and its cap were coated with 1 ml of a solution containing 150 µg/ml clothianidin dissolved in ethanol following the CDC guidelines [53]. For each bioassay test, we used four bottles coated with clothianidin and two control bottles treated with 1 ml of absolute ethanol. All bottles were wrapped in aluminum foil and allowed to dry for 24 h to enable complete evaporation of the solvent before use. Coated bottles were not reused and were washed three times in warm soapy water and allowed to dry for 24 h between experiments. 20 to 25 2–5-day-old females were aspired from mosquito cages and released into one of the test bottles where they were exposed to the active ingredient or into control bottles containing ethanol for 1 h. After the exposure period, mosquitoes were transferred into a paper cup and provided with 10% sugar solution. 60-min knockdown rates measured as the mosquito’s inability to move or fly when touched with forceps were scored, and mortality was monitored every 24 h for seven consecutive days. We used adults from two susceptible strains as controls: *An. gambiae* Kisumu and *An. coluzzii* Ngousso. Both strains are known to be susceptible to pyrethroid, carbamate, organochlorine and organophosphate insecticides.

### Synergist bioassay

We conducted a synergist bioassay to test if piperonyl butoxide (PBO) could enhance the potency of clothianidin. PBO is an inhibitor of oxidases and non-specific esterases involved in metabolic detoxification of insecticides [58]. A solution of 400 µg/ml PBO was prepared by diluting PBO with absolute ethanol. The solution was mixed and stored at 4°C before use. Wheaton bottles and their cap were coated with 1 ml of the PBO solution, wrapped in aluminum foil and allowed to dry for 24 h before the test. 100 to 150 female mosquitoes aged between 2 and 5 days were pre-exposed for 1 h to PBO-coated bottles or to control bottles coated with ethanol. Mosquitoes were then removed from the bottles and batches of 20 to 25 individuals were introduced into new bottles coated with 150 µg/ml clothianidin or with absolute ethanol (control). After 1 h, mosquitoes were transferred into a paper cup and provided with 10% sugar solution. Knockdowns were scored and mortality was monitored every 24 h for seven days. We compared mortality rates with or without the synergist to determine if oxydase inhibition by PBO affected the level of susceptibility.

### Efficacy of SumiShield 50WG

We used WHO tube tests to assess the efficacy of SumiShield 50WG against adult mosquitoes displaying a gradient of tolerance to clothianidin [59]. We used a discriminating dose of 2% w/v clothianidin (13.2 mg active ingredient per paper) following the manufacturer’s recommendation [23, 60]. We prepared a stock solution by diluting 264 mg SumiShield 50WG in 20 ml distilled water. We impregnated Whatman filter papers (12 × 15 cm) containing 13.2 mg clothianidin each using 2 ml of the insecticide solution as described in [61]. Control filter papers were impregnated using 2 ml of distilled water. Treated filter papers were allowed to dry overnight and were kept in foil at 4°C until use. To carry out bioassay tests, we released 20 to 25 2–5-day-old female mosquitoes into each of the test tubes containing clothianidin-impregnated papers. We concomitantly released 20–25 mosquitoes into each control tube. After 1 h, mosquitoes were transferred into holding tubes, and knockdowns were recorded. As with CDC bottle tests, mortality was scored every day until day 7, and mosquitoes were provided with 10% sugar solution.

### Data analysis

We used mortality rates to evaluate the efficacy of clothianidin against laboratory and field mosquitoes. All tests with mortality > 20% at day 7 in controls were discarded. We used Abbott’s formula to correct the mortality rate of the test if 5–20% individuals died between day 1 and day 7 in the corresponding controls [62]. Following the WHO guidelines on insecticide susceptibility, mosquito populations were considered susceptible if mortality at day 7 was ≥ 98% and resistant if mortality was less than 90%. Mortality rates between 90 and 97% implied that the presence of resistant genes in the vector population must be confirmed by additional tests [59]. We used Fisher’s exact test with a significance threshold set at 0.05 to evaluate if mortality rates were significantly different between tested populations. We performed all analyses using the R software (Version 4.2.2) [63].

## Results

### Evidence of clothianidin resistance in *Anopheles gambiae*

**Fig. 3:**
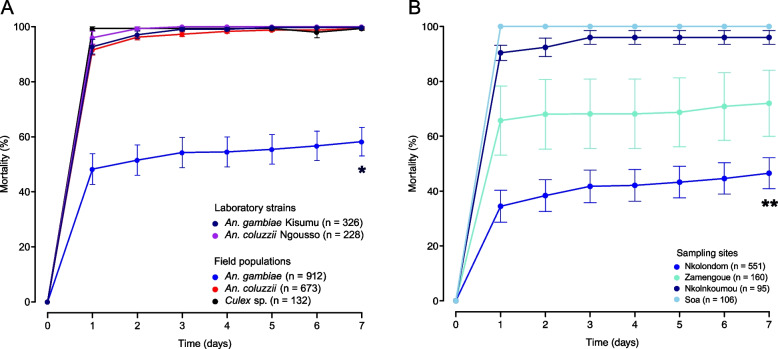
Baseline susceptibility of laboratory strains and field populations to clothianidin monitored for 7 days using CDC bottle bioassays. **A** Mortality values of *Anopheles* and *Culex* female adults exposed to 150 µg/ml of clothianidin. **B** Gradient of susceptibility revealed in wild populations of *An. gambiae*. Error bars represent the standard error of the mean and (n) the number of individuals tested. * Fisher’s exact test (*p* < 0.05) indicates lower mortality in *An. gambiae* compared to *An. coluzzii*. ** Fisher’s exact test (*p* < 0.05) indicates lower mortality in *An. gambiae* from Nkolondom compared to any other conspecific population

**Fig. 4:**
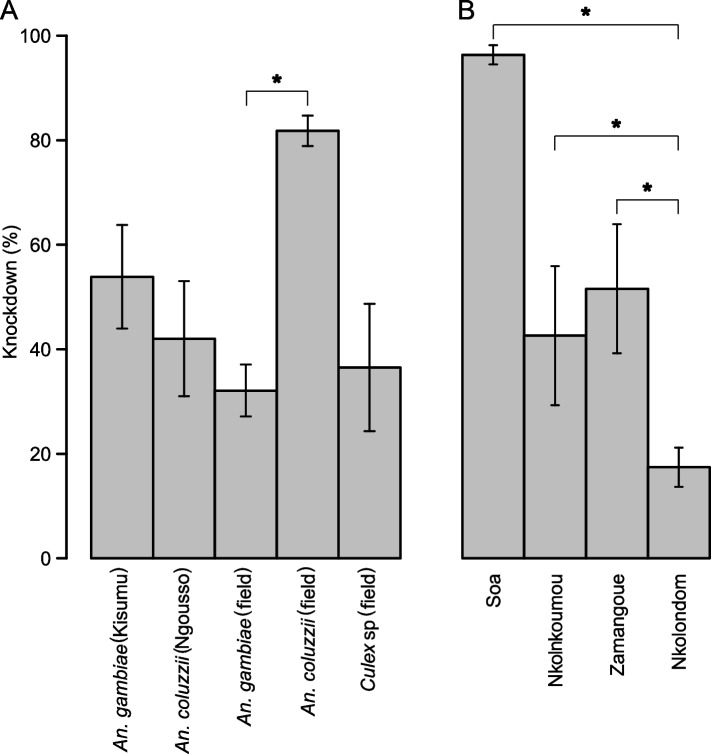
Knockdown values after 1 h exposure to 150 µg/ml of clothianidin in bottle bioassays. Knockdowns were compared between *Anopheles* and *Culex* species (**A**) and between field populations of *An. gambiae* (**B**). Standard errors of the mean are shown as vertical bars. * *p* < 0.05 (Fisher’s exact test)

We used CDC bottle bioassays to test a discriminating dose of clothianidin against a total of 1665 wild mosquitoes belonging to three species: *An. gambiae* (*n* = 912), *An. coluzzii* (*n* = 673) and *Culex* sp (*n* = 132) collected from 6 sampling sites (Table 1). To validate our bioassay protocol, we first analyzed susceptibility in 554 individuals from two laboratory colonies: *An. gambiae* Kisumu (*n* = 326) and *An. coluzzii* Ngousso (*n* = 228). Results showed that the two laboratory strains were fully susceptible to clothianidin, reaching 100% mortality within 1–3 days of exposure (Fig. 3A). Among field mosquitoes, *Culex* sp*.* populations were the most susceptible to clothianidin, reaching 100% mortality approximately 24 h post exposure (Fig. 3A). A diagnostic PCR confirmed that specimens collected from the two urbanized sites were 100% *An. coluzzii*. These *An. coluzzii* populations were also susceptible to clothianidin, but 100% mortality was reached between the second and the fourth day. In *An. gambiae* by contrast, the overall mortality in all 817 individuals tested from 4 locations was only 58.2 ± 5.2%, suggesting that some populations of this species have developed resistance to clothianidin. Mortality was significantly lower in *An. gambiae* compared to *An. coluzzii* (*p* < 2.2e-16, Fisher’s exact test). Knockdown at 60 min was generally low, except in field populations of *An. coluzzii* (81.8 ± 2.9%). Knockdown within 60 min of exposure ranged from 42 ± 11.0% to 53.9 ± 9.9% between lab strains and from 32.1 ± 5.0% to 36.5 ± 12.2% among field populations (Fig. 4A). Consistent with their reduced susceptibility to clothianidin, knockdown was significantly lower in wild populations of *An. gambiae* (32.1 ± 5.0%) compared to *An. coluzzii* (81.8 ± 2.9%) (*p* < 2.2e-16, Fisher’s exact test) (Fig. 4A).

**Table 1:** Description of the six sampling sites and adult mosquito populations tested

**Species**	**Population**	**Site**				**Sample size**	**Bottle bioassay**	**WHO tube test**	**PBO synergist**
		**Name**	**Classification**	**Latitude**	**Longitude**				
*An. gambiae*	Lab strain	Kisumu				326	326	0	0
	Field	Nkolondom	Suburban	3°95′54''N	11°49′74''E	822	551	171	100
		Nkolnkoumou	Suburban	3°86′02''N	11°39′42''E	195	95	100	0
		Zamengoue	Suburban	3°94′52''N	11°45′02''E	160	160	0	0
		Soa	Suburban	3°99′55''N	11°59′84''E	106	106	0	0
*An. coluzzii*	Lab strain	Ngousso				328	228	100	0
	Field	Combattant	Urban	3°88′30''N	11°51′08''E	710	483	227	0
		Tsinga	Urban	3°80′46''N	11°50′06''E	190	190	100	0
*Culex sp.*	Field	Nkolondom	Suburban	3°95′54''N	11°49′74''E	132	132	100	0

### Resistance is stronger in populations from the agricultural area

To test if susceptibility to clothianidin varies across geographic areas*,* we compared the profiles of *An. gambiae* adult populations collected from four different sites. Based on the diagnostic PCR, larvae from the farm (Nkolondom) and from one of the suburban sites (Zamengoue) were 100% *An. gambiae*. Samples from the two remaining sub-urban sites, Soa and Nkolnkoumou, were ~ 80% *An. gambiae*/20% *An. coluzzii*. The four sites were treated as *An. gambiae* habitats. Mortality rates varied along a geographic gradient, ranging from susceptibility in Soa and Nkolnkoumou to resistance in samples originating from the agricultural site, Nkolondom. Only 46.5 ± 5.7% of individuals from the farm died between the first and the seventh day post-exposure (Fig. 3B). Mortality of adults from Nkolondom was significantly lower compared to samples from Zamengoue (*p* = 1.60e-11, Fisher’s exact test), from Nkolnkoumou (*p* < 2.2e-16) and from Soa (*p* < 2.2e-16). Knockdown at 60 min was also significantly lower (20.1 ± 4.1%) in samples from the agricultural site compared to the other *An. gambiae* populations (*p* < 0.05, Fisher’s exact test) (Fig. 4B).

### PBO is a synergist of clothianidin

**Fig. 5:**
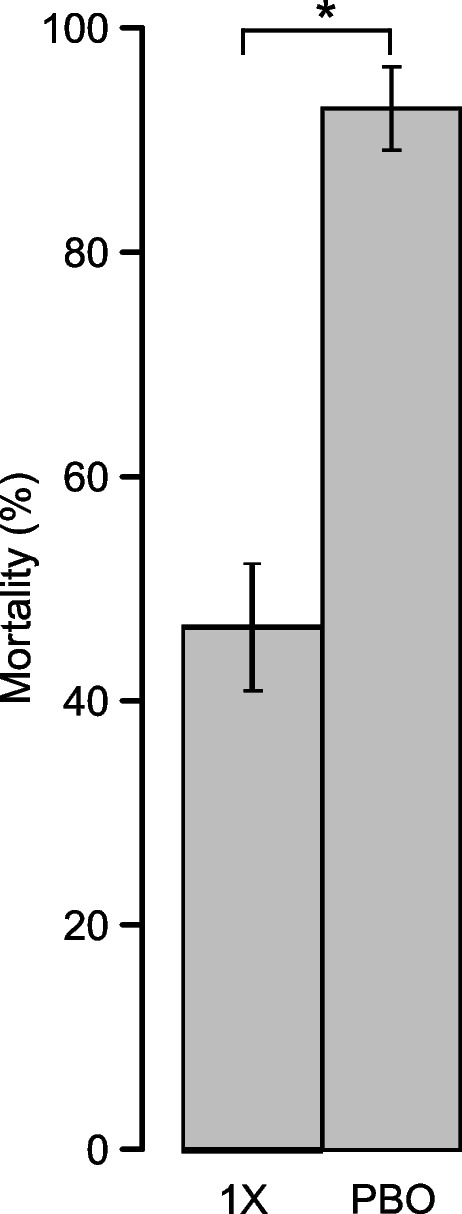
Synergistic effect of PBO. A standard test with 150 µg/ml of clothianidin (1X) and a synergist bioassay (PBO) showed a significant increase in mortality in the presence of PBO. Error bars represent the standard error of the mean. * *p* < 0.05 (Fisher’s exact test)

Mortality observed in clothianidin-resistant populations of *An. gambiae* from the agricultural site increased from 46.5 ± 5.7% without PBO to 92.7 ± 3.7% when adult mosquitoes were pre-exposed to the synergist (*p* = 6.08e-10, Fisher’s exact test) (Fig. 5). This result suggested that metabolic detoxification involving cytochrome P450 enzymes contributes to the development of resistance to clothianidin in *An. gambiae*.

### Resistance to clothianidin reduces the efficacy of SumiShield 50WG

**Fig. 6:**
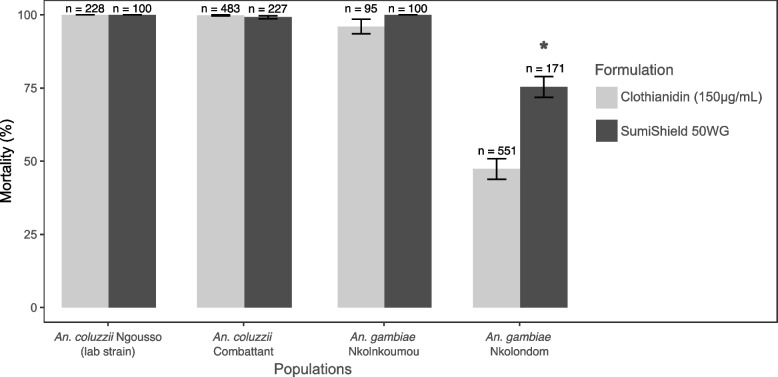
Relationship between susceptibility to clothianidin as revealed by CDC bottle bioassays and efficacy of SumiShield 50WG evaluated with WHO tube tests. Any population susceptible to clothianidin (150 µg/ml) reached 100% mortality within 3 days of exposure to SumiShield 50WG. Error bars represent the standard error of the mean. * Fisher’s exact test (*p* < 0.05) indicates lower mortality in *An. gambiae* from Nkolondom compared to any other population tested with SumiShield 50WG

To ascertain to what extent clothianidin resistance could impact the efficacy of manufactured formulations, we used WHO tube tests to estimate the susceptibility of young female adult mosquitoes to SumiShield 50WG. This formulation corresponds to ~ 25-fold the discriminating dose of clothianidin used in CDC bottle assays. We tested one *An. coluzzii* population from the urban area (Combattant), one susceptible *An. gambiae* population (Nkolnkoumou) and resistant mosquitoes from Nkolondom. We used the laboratory strain *An. coluzzii* Ngousso as negative control. As expected, the lab strain was susceptible to SumiShield 50WG, reaching 100% within 48 h of exposure to the formulation (Fig. 6). Among the field populations, susceptibility to clothianidin as revealed by bottle bioassays was a good predictor of the efficacy of SumiShield 50WG. Both *An. coluzzii* and *An. gambiae* populations that were fully susceptible to the active ingredient were also susceptible to the IRS formulation. By contrast, *An. gambiae* populations from Nkolondom that were resistant to 150 µg/ml clothianidin in bottle bioassays were less susceptible to SumiShield 50WG. Mortality rates against filter papers impregnated with SumiShield 50WG at a diagnostic dose of 2% (w/v) clothianidin was only 75.4 ± 3.5% after 7 days of holding period and was significantly lower compared to *An. coluzzii* Ngousso (*p* = 9.08e-5, Fisher’s exact test), *An. coluzzii* Combattant (*p* = 5.91e-15) and *An. gambiae* Nkolnkoumou (*p* = 1.22e-7). This finding suggests that *Anopheles* populations with low mortality to 150 µg/ml clothianidin have the potential to develop resistance to some neonicotinoid formulations used for indoor residual spraying.

## Discussion

Widespread pyrethroid resistance has been associated with a decline in the efficacy of LLINs and IRS in several countries [64–66]. Here we have shown that some new active ingredients may also have reduced efficacy against some vector populations. In *An. gambiae* mosquitoes from Yaoundé, the efficacy of SumiShield 50WG, a formulation of clothianidin prequalified for IRS is reduced in populations that have evolved resistance to the active ingredient.

We first evaluated the susceptibility of *Anopheles* and *Culex* mosquitoes to clothianidin using CDC bottle bioassays. We used a protocol that differed slightly from the WHO standard operating procedure for testing the susceptibility of adult mosquitoes to clothianidin [54, 67]. We made the choice not to use a vegetable oil ester (Mero) as a surfactant as suggested by the standard operating procedure because it has been shown that this adjuvant can have synergistic interactions with neonicotinoids [34, 43, 56]. Additionally, at a discriminating concentration of 150 µg/ml, there was no need to add a surfactant to increase solubility because clothianidin was soluble in ethanol when the mixture was allowed to rest for at least 24 h before use.

Using a discriminating concentration of 150 µg/ml in CDC bottle bioassays, we detected resistance to clothianidin in wild *An. gambiae* mosquitoes. Knockdowns after 1 h exposure to the insecticide were low and had little discriminative power. Other studies assessing the susceptibility of adults *Anopheles* mosquitoes to clothianidin have reported low knockdowns, which could be due to the fact that clothianidin act slowly compared to other neurotoxic insecticides such as pyrethroids [17, 23, 57, 60]. Contrary to knockdowns, monitoring mortality rates for seven days provided a reliable measure of susceptibility to clothianidin in adult mosquitoes. We first observed that susceptibility vary between species. *An. coluzzii* adults collected from urban areas of Yaoundé were susceptible to clothianidin. As crop cultivation associated with neonicotinoid spraying is less frequent in urban areas, *An. coluzzii* larvae from urbanized settings in Yaoundé are presumably less exposed to neonicotinoids residues and are in theory less likely to develop resistance to neonicotinoids. In *An. gambiae* however, the situation was more complex, with a gradient of susceptibility to clothianidin established among suburban and rural populations. Populations from a farm where neonicotinoids are used weekly for crop protection were the most resistant to clothianidin (Fig. 2B). Indeed, during our field survey in the farm, we collected empty containers of imidacloprid and acetamiprid confirming their use (Fig. 2B). Dozens of formulations of the two insecticides are freely sold in local stores in Yaoundé and are intensively applied by farmers [39, 42]. The results presented in the current study are based on field surveys that were conducted between 2019 and 2020. The findings have been supported by monitoring that continues from 2020 to 2022 and confirmed patterns of susceptibility to clothianidin observed in precedent years in *Anopheles* mosquitoes from Yaoundé and its neighboring rural areas [33]. These surveys have combined larval tests and adult bioassays to reveal that neonicotinoid resistance is emerging in *An. gambiae* populations from the equatorial forest region of Cameroon, especially in areas where larvae are chronically exposed to pesticide residues [32–34]. There is ample evidence that *An. gambiae* larvae and adults from several villages around the city of Yaoundé are currently resistant to imidacloprid, acetamiprid and thiamethoxam, three neonicotinoids that are among the most widely used crop protection chemicals in Cameroon [32, 33, 39, 42]. A study conducted in Ivory Coast has also observed resistance to imidacloprid and acetamiprid in *An. coluzzii* correlated with agricultural activities [35].

Intriguingly, adults of *Culex sp* whose larvae were collected from the same breeding sites as *An. gambiae* in Nkolondom were fully susceptible to clothianidin. However, it is well known that aquatic invertebrates have variable responses and threshold of susceptibility to the lethal and sublethal effects caused by neonicotinoid contaminants [68]. Although *Culex* sp populations were more directly impacted by the lethal toxicity of clothianidin, they likely have developed other physiological and/or behavioral adjustments enabling them to adapt to neonicotinoid residues in farms [69, 70].

Bioassay tests using CDC bottles coated with the synergist PBO prior to exposure to clothianidin showed a drastic increase in mortality in resistant populations. This suggested that Cytochrome P450 enzymes (CYPs) play a primarily role in neonicotinoid resistance in *An. gambiae*. Acetamiprid resistance in *An. gambiae* is also highly dependent on metabolic detoxification mediated by CYPs [33]. More generally, neonicotinoid resistance in wild populations of many crop pests, primarily those of the order *Hemiptera* (aphids, whiteflies, and planthoppers), is associated with overexpression of one or several CYP enzymes [71–74].

The spread of pyrethroid resistance has caused a decline in the effectiveness of LIINs and IRS [64, 65]. Resistance could also undermine the efficacy of new products such as SumiShield 50WG and Fludora Fusion. Larval bioassays showed that the intensity of resistance to clothianidin in *An. gambiae* from Nkolondom is currently similar to that of deltamethrin [32]. Therefore, even without any large-scale deployment of clothianidin in vector control, its efficacy may already be as reduced as that of pyrethroids in some populations [75]. In our study, we have revealed that the efficacy of SumiShield 50WG is declining in clothianidin-resistant populations. We did not test Fludora Fusion or 2GARD, and it remains to be evaluated if the dual action of clothianidin and deltamethrin will result in higher efficacy against resistant populations. However, a recent experiment has demonstrated that exposure of *An. gambiae* larvae to sublethal doses of a mixture of different types of agrochemicals increased the tolerance of adults to both clothianidin and Fludora Fusion [76]. This suggested that the efficacy of this formulation could also be impacted if clothianidin resistance spreads among anopheline populations.

Some caveats in the interpretation of findings from the current study need to be highlighted. Although resistance to clothianidin was detected in *An. gambiae* populations from an agricultural area, it is difficult to draw robust conclusions about the role of agriculture in the development of resistance given the relatively small geographic extent of the study. In addition, the contrasting level of tolerance to neonicotinoids observed between the sibling species *An. gambiae* and *An. coluzzii* suggests that the species factor might have a stronger explanatory power than the environmental pressure. In line with this prediction some *An. funestus* populations whose larval populations thrive in waters that are presumably less likely to be contaminated with pesticide residues can tolerate 150 µg/ml of clothianidin [43]. Overexpression of cytochrome P450 enzymes (CYPs) is a prime mechanism used by *An. funestus* populations to resist to several classes of insecticides. It is likely that some CYPs contribute to the degradation of clothianidin and to reducing the susceptibility to neonicotinoids. Further research is needed to better understand the contribution of genetic and environmental factors in the development of neonicotinoid resistance in *Anopheles* species. Here, using a standard synergist test, we showed that cytochrome P 450s likely play an important role in clothianidin resistance in *An. gambiae*. However, the role of other mechanisms such as target-site mutations, cuticle resistance and behavioral shifts have yet to be elucidated.

## Conclusions

The current study shows that the new insecticide clothianidin may have reduced efficacy in some areas due to pre-existing levels of resistance among mosquito populations. These findings suggest that prior to inclusion of agrochemicals in resistance management programs, variation in susceptibility among vector species as well as cross-resistance due to residual pesticide exposure and/or to the ubiquitous activity of some detoxification enzymes should be particularly scrutinized.

## Data Availability

The data for this study have been presented within this article.
